# Grape Seed Oil as a Natural Therapy in Male Rats with Alzheimer’s Diseases

**DOI:** 10.34172/apb.2020.052

**Published:** 2020-05-11

**Authors:** Farnoosh Berahmand, Golnoush Anoush, Mir-Jamal Hosseini, Mahdieh Anoush

**Affiliations:** ^1^Student’s Research Center, Zanjan University of Medical Sciences, Zanjan, 4513956184, Iran.; ^2^Department of Toxicology, School of Pharmacy, Azad University of Shahreza, Isfahan, 8614510311, Iran.; ^3^Applied Pharmacology Research Center, School of Pharmacy, Zanjan University of Medical Sciences, Zanjan, 4513956184, Iran.; ^4^Department of Pharmacology, School of Pharmacy, Zanjan University of Medical Sciences, Zanjan, 4513956184, Iran.

**Keywords:** Acetylcholine, Alzheimer’s disease, Grape seed oil, Rat, Scopolamine, Spatial memory

## Abstract

***Purpose:*** Alzheimer’s disease (AD) is a chronic neurodegenerative disorder, with an increasing prevalence rate, mostly related to cholinergic system. According to the difficulties and complications in management of AD, this study was carried out to evaluate the efficacy of grape seed oil (GSO) on scopolamine (Scop) induced Alzheimer’s in male rats.

***Methods:*** 64 healthy male Wistar rats received different treatments such as: normal saline (NS), donepezil (Don), Scop and GSO, according to the previously designed protocol. Morris (MWM) was applied for spatial memory tests. Right after the behavioral tests, the brains were removed and the hippocampus was separated for evaluation of acetylcholine levels as well as cell death and neuro inflammation.

***Results:*** The results of the test day indicated that the mean Q2 time was increased in both GSO test groups (*P* <0.05) and Don treated group (*P* <0.001).The spectrophotometric findings affirm that both GSO co-treatment and post-treatment were effective in augmenting brain acetylcholine levels (*P* <0.01 and *P* <0.05 respectively). The microscopic findings of H&E dyed tissues confirmed the above mentioned results for different treatments except for GSO post treatment, in which the viability of cells were very low.

***Conclusion:*** The results implied that supplementation of rats with GSO caused a significant augmentation in spatial memory performance as well as acetylcholine levels and cell viability in the presence of Scop. This effect was comparable to that of Don especially when GSO was used as co-treatment.

## Introduction

Alzheimer’s disease (AD) is the most frequent form of dementia with more than 36 million people suffering from, around the world with an increasing rate which make AD a very serious health issue.^1,2^ The manifestations of this neurodegenerative disease include behavioral and neuropsychiatric symptoms, cognitive decline, memory loss and impaired activities of daily living. From pathological point of view, formation of neuritic plaques and neurofibrillary tangles in brain results in AD.^3^ This leads to impaired synaptic functions and structures, progressive deposition of tau protein, neuronal destruction and ultimately the clinical manifestations of AD.^4^ Both the cholinergic and glutamatergic neurotransmission systems which have been proven to play key roles in cognition, are affected in AD patients; specially a disturbed cholinergic system is responsible for deterioration in memory and cognition in patients with AD.^5^ Besides, oxidative stress is known as a cause and therapeutic goal of AD. Increased reactive oxygen species (ROS) are capable of damaging proteins, lipids and neurons at the final stage. On the other hand, neuro-inflammation can contribute to progressive neurodegeneration.^6,7^ In this context, the drugs which target various pathways such as cholinergic and glutamatergic systems are applied in order to restore the impaired function of neuron.^8^ Evidence shows the role of acetylcholine in reducing the production of inflammatory cytokines. Therefore, a reduced amount of acetylcholine in the brain during AD, can increase inflammation and exacerbate the disease.^9^ Besides, Increased level of acetylcholine in the brain may improve cognitive ability.^10^

Centrally-acting drugs that inhibit acetylcholinesterase and thus reduce acetylcholine degradation can be a suitable way to control AD progression.^11^ So, a selective therapeutic option is to increase the level of acetylcholine by using cholinesterase inhibitors such as donepezil (Don) which reduces synaptic acetylcholine degradation and enhances the normal pattern of acetylcholine release in the brain.^12^ Drugs like Don and galantamine are able to activate the cholinergic anti-inflammatory pathway.^9^

Natural products, according to their various compounds, can be beneficial through diverse mechanisms like oxidative stress inhibition, which reduces ROS formation and results in inhibition of bio-molecule’s oxidation and cellular damage.^13^ This pathway can consequently suppresses neurodegenerative diseases.^14^ Therefore, treatment with antioxidants is a promising approach for reducing disease progression.^15^

The current research has found a link between components of a widely used natural oil and reduction of dementia rate.

Grape seeds are a rich source of phytosterols and polyphenol.^16,17^ Their oil contains different kinds of saturated and unsaturated fatty acid including linoleic (65%), linolenic (1.5%), oleic (17%), palmitic (8.0%), stearic (4.4%) and arachidonic (0.6%) acids. Linoleic acid (C18:2) and oleic acid (C18:1) are most important unsaturated fatty acids present in grape seed oil (GSO).^18^

Many possible mechanisms have been proposed for the grape seeds’ neuroprotective effects such as free radical scavenging via reducing lipid peroxidation, exerting superior antioxidant efficacy, inhibiting DNA oxidative and blocking cell death signaling.^19,20^ Grape seed extract demonstrated to be effective on memory remission in beta amyloid induced memory loss,^21^ but such a study has not been done on GSO. So, the aim of the present study is to evaluate the possible effects of GSO on AD model in male rats.

## Materials and Methods

### Animals

Sixty-four male Wistar rats (150-175 g) were purchased from Pasteur Institute, Tehran, Iran. The animals were kept in standard condition including 12:12 h light/dark cycle at 21-23°C and were allowed access to food and water *ad libitum* in social condition.

### Chemicals

Donepezil, Scopolamine Hydro bromide, Acetylcholine Chloride and Acetylthiocholine Iodide, were purchased from Sigma-Aldrich (Sigma-Aldrich Chemie, GmbH, Heimstadt, Germany). Grape seed oil which was produced via cold press method from seeds of Vitis Viniferra prepared from Natural Sourcing, LLC. Hydroxylamine hydrochloride, Sodium Hydroxide, HCL, Ferric Chloride, Sodium Acetate, Formic acid, and pure acetone purchased from Merc, Germany.

### Experimental design

The animals were randomly divided into 7 groups of 8 rats in each, and received different treatments as described below:

- Group 1 (NS group): The healthy rats in this group received intraperitoneal (IP) injection of 1ml.kg-1 normal saline (NS) as the solvent for scopolamine (Scop) for 10 consecutive days.
- Group 2, AD control group (Scop): This group received Scop (1 mg.kg-1 in NS, IP) for 10 consecutive days.
- Group 3 (Don group): The rats were administered IP injection of Don at 1 mg/kg dosage for 10 consecutive days.
- Group 4 (GSO group): They received GSO 2 mL/kg by gavage for 10 consecutive days.
- Group 5, Scop received group with Don post treatment (Scop/Don): This group received Scop (1 mg/kg in NS, IP) for 10 days followed by Don administration for another 10 days (1 mg/kg)
- Group 6, Scop received rats with GSO post treatment (Scop/GSO), which administered Scop (1 mg/kg in NS, IP) for 10 days followed by GSO gavage (2 mL/kg) for another 10 days.
- Group 7, Don co-treated Scop received group (Don/Scop), which received Don (1 mg/kg) and Scop (1 mg/kg in NS, IP) respectively, in the same day with a 30 minutes time interval; for 10 days.
- Group 8, GSO co-treated Scop received group (GSO/Scop), which administered GSO gavage (2 mL/kg) and Scop (1 mg/kg in NS, IP) respectively, in the same day with a 30 minutes time interval; for 10 days.

Training days in MWM started right after the final day of treatments for each group. At the end of the behavioral experiments; the animals were fully anesthetized applying IP injection of 5 mL/kg of ketamine/Xylazine and then decapitated using a sharped blade guillotine in order to exert brain tissue. The exerted brains used for evaluation of histopathological changes as well as acetylcholine levels analysis.

### Alzheimer’s induction in rats

After dissolving Scop (Sigma, St Louis, MO, USA) in NS (0.9%), intraperitoneal injection of Scop (1 mg/kg) was administered to animal, based on previous studies.^22^ Behavioral assessment and histopathological findings on the last day, demonstrated the affected learning and memory behaviors as well as cell death and tissue viability qualification.

### Behavioral assessment

Morris water maze (MWM) behavioral test is used to assess the spatial learning memory ability as described fully by D’Hooge and De Deyn^23^ in 2001. The animals were placed gently in a large round black pool (150× 60×30 cm) filled with water. The hidden and submerged escape platform is placed 1cm below-water surface in a specified zone of pool according to the attached software. Animals were trained for 4 consecutive days in the initial learning phase to learn the place of the platform and try to find it. A cut-of-time of 60 seconds was selected for each rat to swim inside the pool. The rats that failed to find the platform in 60 seconds, were guided by investigator to find the platform after the software stopped the run. At the end of each run, the rats were wiped and put under a heather. On the test day (the 5^th^ day), the platform was removed and rats were given a 60 seconds trial time to find the platform.^23^ Spatial memory retention on the test day, can be determined by the percentage of time animals spent within the “target” quadrant of the pool that previously contained the hidden escape platform.

### Histopathological study

Animals were decapitated and the brain was isolated and immersed in 10% formalin, dehydrated in alcohols and embedded in paraffin. Slides of hippocampus tissue were stained using hematoxylin and eosin (H&E) for general histopathological examination in according to standard protocols and the CA1 region observed with an optical microscope for qualitative evaluation of necrosis and possible neuronal damages.

### Acetylcholine level assay in brain 

After decapitation of animals, the brains were exerted and kept at -80°C. In order to evaluating the acetylcholine amounts according to Hestrin and Stepankova^24^; the frozen brains were first powdered with dry liquid nitrogen in the mortar. 150 mg of powdered tissue was mixed with 2 mL of a mixture of formic acid and acetone (15%v/v) and kept in ice for 30 minutes, then centrifuged for 5 minutes at 950 g. After separating the supernatant, the precipitate was mixed with 5.0 mL of formic acid-acetone 70% and again, centrifuged in 0°C. Ultimately, supernatants were collected and the Ach amounts were calculated on the basis of calibration curve equilibrium using a spectrophotometer at 540 nm.

### Statistical analysis

Statistical analyses were performed by SPSS, version17. Data were expressed as mean ± standard error of means (SEM). Comparisons between the groups were made using one-way analysis of variance (ANOVA), followed by Tukey’s post hoc test. *P* values less than 0.05 were considered statistically significant.

## Results

### Spatial learning memory function in water maze assay

**Figure 1:**
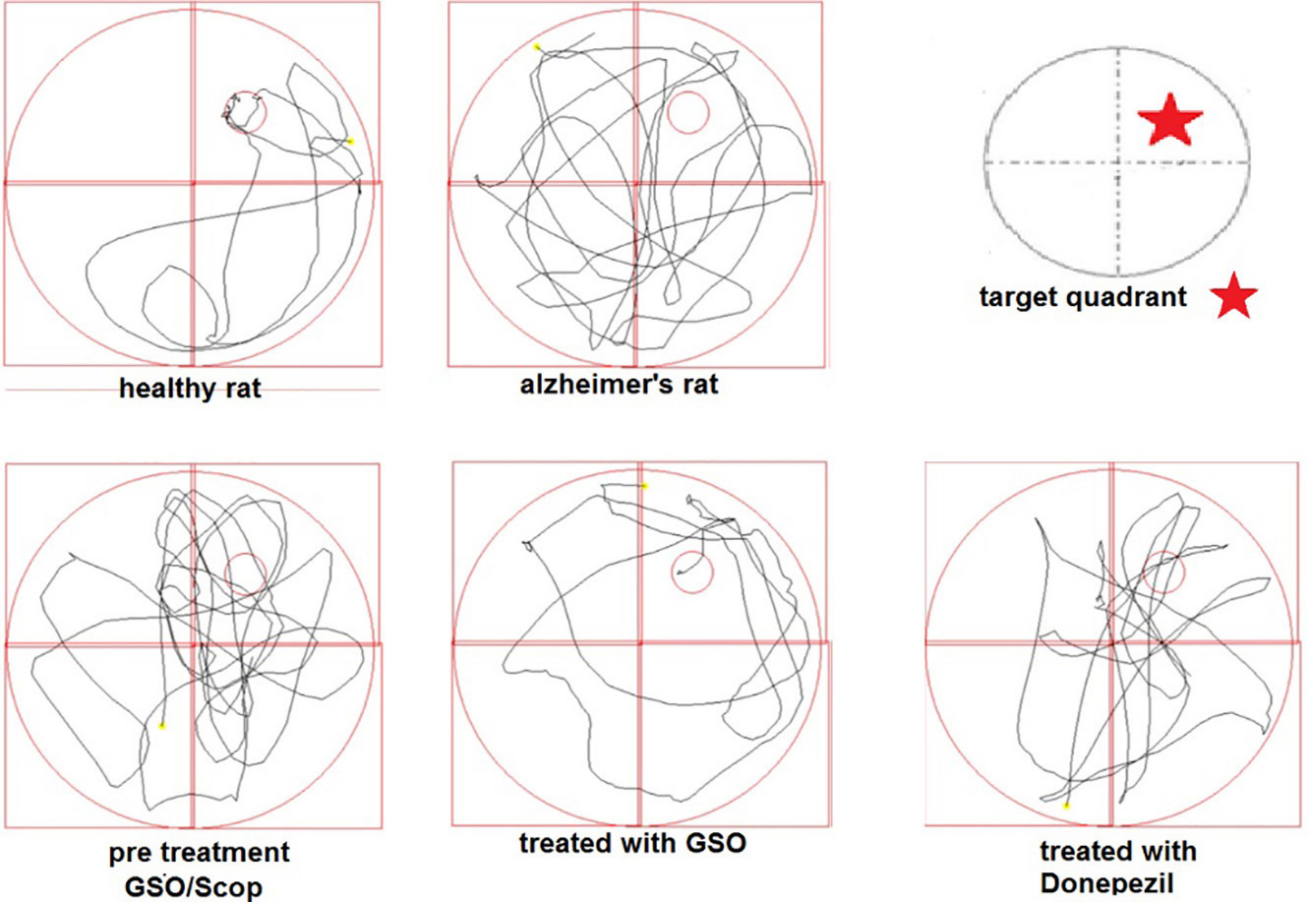
The sample swimming pathways of rats in water maze on test day.
The swimming path for some of the rats on the test day is shown in Figure 1. As it is shown, healthy rats, rats received preventive grape seed oil and the scopolamine received rats which were treated with Don; have spent most of their time in the quarter of target, while rats received scopolamine spent almost a similar time in every quarter with a round shaped path.

**Figure 2:**
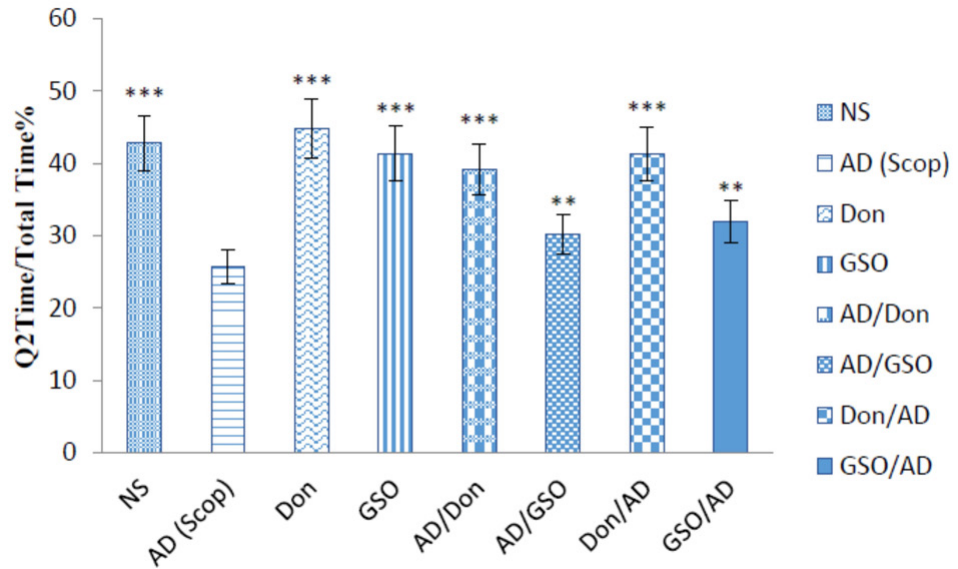
Q2 Time / total time percentage in all groups on test day.
There were 8 rats in each group. Data are presented as mean ± SEM. All groups were compared to Scop (scopolamine received) group. **P < 0.01; *** *P* <0.001.

Figure 1 demonstrates some random swimming patterns of animals from 4 important groups. The swimming pathways show that Figure 2 shows the final effects of treatments on the test day. All rats were tested on day 5. The behavioral results show not only a better learning pattern for treated animals either with Don, or with GSO; but also a significant difference between the Scop received group and GSO pre/post treated groups (*P* < 0.01).

Control groups which received NS, Don and GSO, did not differ significantly with each other in the time spent in Q2, and the time for all of them were higher than that of Scop received group (*P* < 0.001). In other words, receiving Don or GSO in healthy rats did not affect the memory and made it better in the presence of Scop (Figure 2).

On the other hand, co-treatment with Don and GSO demonstrates a significant increase in the Q2 time percentage. Both the groups treated and pretreated with GSO demonstrated a significant difference with AD group but were not as effective as Don. Therefore, it can be deducted that GSO could be effective in reducing or preventing the destructive effects of Scop on the cholinergic system (Figure 2).

### The brain acetylcholine levels

The acetylcholine concentration in animals’ brain was measured by Hestrin method and the final spectrophotometry results were recorded and put into the equivalent formula obtained from calibration curve for the standard solution of acetylthiocholine.^25^

**Figure 3:**
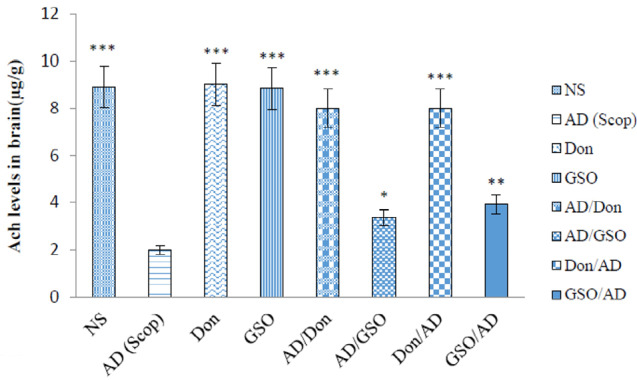
Comparison between acetylcholine levels in all groups.
Six brain samples were prepared for each group and all the detections were repeated 3times for each sample. Data are presented as the mean ± SEM. All groups were compared to Scopolamine received group. *P <0.05 , **P < 0.01; *** *P* <0.001.

According to Figure 3, the lowest level of Ach was observed in the group received Scop followed by groups administered GSO after Scop and before Scop; in which the levels of Ach were significantly higher than the control (AD) group (*P* < 0.05 and *P* < 0.01 respectively). All the other groups had a reasonably higher Ach levels (*P* < 0.001) in comparison with control group (Figure 3).

By comparing the mean concentrations of acetylcholine in the group received GSO as a preventative with other groups, it is clear that although this group had a significant difference with other groups (*P* < 0.05), but in turn differs from the AD group (*P* < 0.05). In the group which received GSO before and at the same time with Scop, the results indicated that GSO was capable of both preventing the destruction of the acetylcholine induced by Scop and reducing its effects, but the preventive effect was stronger than that of the treating effect. Both co-treatment and post-treatment with Don resulted in a high amount of Ach levels compared to AD group which received Scop (*P* < 0.001).

### Histopathological results

**Figure 4:**
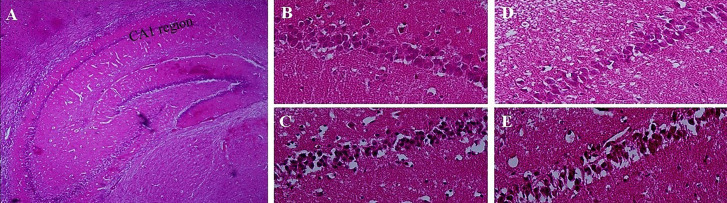
Histopathological changes of CA1 region in brain tissues.
The extracted brain tissues were prepared for H&E dying method and the pictures were selected randomly from CA1 regions among eight samples of each group. (A) An image with 40 magnification demonstrated CA1 region. (B) Normal saline received group. (C) Scopolamine received (AD) group. (D)GSO/Scop group. (E) Scop/GSO rats. The images B- E (with400 magnification) demonstrated CA1 region. The normal cells and tissue consistency as visible in Band D, while cell death and inconsistency is apparent in images C and E.

Figure 4 demonstrates the histopathologicalchanges in CA1 region of hippocampus of rats’ brain tissues in the 4 major groups. Figure 4A demonstrated the observed region of hippocampus in all groups (i.e. CA1). Figure 4B shows the normal cells and consistent tissue of a rat received NS, (Figure 4B). In the Scop received animals, neuronal degeneration along with the vacuolization of the nerve tissue were observed. Besides, the wrinkling and dense nuclei are indicatives of necrotic neurons (Figure 4C). The CA1 region In the GSO received group demonstrates very low neuronal rupture and tissue degradation (Figure 4D) which is similar to the characteristics of the control brain (Figure 4B). Nevertheless, the image from CA1region in the AD group, post treated with GSO (Figure 4E), is almost similar to Figure 4C as presumed, which means that GSO while administered after Scop, was not capable of restoring Scop’s tissue damage aftermath.

## Discussion

This study investigated the effect of GSO on the function of spatial memory in an Alzheimer’s model induced by Scop. In addition, this study evaluated the effects of GSO on acetylcholine levels in rats’ brain. According to the results, the rats which received GSO in both two test groups (GSO/AD and AD/GSO), were tend to pass more time in the quarter with the platform (Figure 2). The hydroxylamine and spectrophotometry results in acetylcholine measurement also confirmed this results.

The histopathological qualitative data obtained from H&E stained brain samples, demonstrated a visible necrosis along with the vacuolization of the nerve tissue which confirmed the ability of Scop to induce apoptosis and necrosis (Figure 4C). Co-treatment with GSO was capable of preventing tissue damage by Scop (Figure 4D). Although, as it was expected and in contrary to the behavioral data, post treatment with GSO was not able to repair the damaged tissue (Figure 4E).

Scop-induced dementia has been used extensively to assess potential therapeutic agents for treating AD.^22^ Scop is a nonselective muscarinic cholinergic receptor antagonist results in cholinergic dysfunction and consequently performance deficits in learning and memory.^26^ In particular, Scop impairs spatial learning and memory in MWM performance and is related to reductions in ACh and increased AChE activity in the hippocampus.^27,28^ Therefore, in this study, Scop was administered to induce AD.

The standard drug was used in the present study, was Don, At present, one of the mostly prescribed treatments for AD as an Ach esterase inhibitor which increases the availability of acetylcholine at cholinergic synapses.^29^ The results of this study showed a potent effect of this drug in both test groups (Don/AD and AD/Don) on Scop induced AD treatment in all three fields that were studied (Figures 2 to 4). The obtained results are in consistency with the results of a study performed by Lian et al showed that Don group exhibited significantly decreased latency (*P* <0.01) and a significantly increased number of platform crossings (*P* <0.01), as compared with non-treated group.^30^

Cognitive impairment of Scop is a result of destruction in cholinergic neurotransmission and increase of oxidative stress.^31^ Thus, factors which are able to ameliorate these effects on cholinergic system and/or oxidative stress might be beneficial in AD treatment. In this study, GSO has been chosen. Our results showed that this agent can be a preventive, and not curative agent in AD.

Normal cellular function require the balance between ROS production and the antioxidant defense. This balance is distrusted in AD altered and the overproduction of ROS combined with the insufficient antioxidant defense leads to oxidative stress.^32^ Evidences shows that increased production of ROS following the mitochondrial damage contributes to the early stages of AD,^33^ in this Regard, the antioxidant effects of some agents like garlic extract, curcumin, melatonin, resveratrol, *Ginkgo biloba* extract, green tea and C and E vitamins in AD patients have been reviewed.^34^

The impact of fatty acid on brain structure and function is clear. One of the important problem in preserving memory and cognitive functions in AD patient is maintenance of the connective brain structure. Unsaturated fatty acid-enriched diet, especially DHA and EPA, can have such effects by influencing on hippocampal atrophy and increased lesions in white material. The epidemiological studies have shown that patients which use the diets rich in these fatty acids are less likely to develop AD resulting from neurodegenerative processes or functional and cognitive loss.^35^

Besides, GSO contains vitamin E (tocopherols and tocotrienols), sterols, phenolic and other bioactive compounds, which all have antioxidant properties.^36,37^ Vitamin E also possesses neuroprotective.^38^

For this reason, the use of GSOs has been suggested to delay the aging process and many practitioners have added Vitamin E supplements to the standard treatment regimen of AD.

One of the other lipophilic compound groups largely found in GSO, are phytosterols, which may prevent the release of pro-inflammatory mediators.^39,40^

A study showed that, the learning and memory process in both GSO received test groups are faster in comparison with the Scop received group during the training days (Data not included). Besides, the percent of time that rats spent in goal quarter during final (trial) day was increased significantly in both GSO/AD and AD/GSO groups. These results imply that supplementation of rats with GSO will be effective in improving spatial memory performance (Figure 2). These findings have indicated that GSO enhanced spatial memory in Scop received male rats thereby protecting the central nervous system from the memory impairment.

These results are in agreement with Bickford et al, which reported that chronic administrations of antioxidants alleviate age-associated cognitive deficits in animals.^41^

In this study, GSO seemed to be able to prevent the necrosis of cells in different sections of hippocampus compared with Scop. (Figure 4). Two suggested pathological mechanisms of AD are formation of extracellular protein assemblies including amyloid beta and formation of Tau plaques. Most drug discovery attempts are intended to prevent or improve the clearance of these pathological assembly. This might be due to high percentage of unsaturated fatty acids which have the ability to reach the brain. They do this by modulating multiple mechanisms including reducing the formation of beta amyloid and also reducing its oligomerization. As this data was not alongside the behavioral and Ach data for the GSO treated Group, we can conclude that there are other routs than neuro-inflammation for GSO to show its effects as discussed above.

## Conclusion

In conclusion, based on the results of the Morris water maze tests, and the measurement of acetylcholine by hydroxylamine method, we found that GSO had remarkable cognitive-enhancing activity by preventing the deleterious effect of Scop especially on Ach levels in male rats.

## Ethical Issues

This study was conducted in accordance with the National Institutes of Health (NIH) Guide for the Care and Use of Laboratory Animals. It was also approved by the Animal Ethics Committee of Zanjan University of Medical Sciences. The ethical approval code was: ZUMS.REC.1396.228.

## Conflict of Interest

Authors declare no conflict of interest in this study.

## Acknowledgments

This research is supported by Research Affairs of Zanjan University of Medical Sciences (ZUMS) and is a part of Pharm.D student thesis of Farnoosh Berahmand which was supported financially by the vice chancellor for research, Zanjan University of Medical Sciences, Zanjan, Iran (the grant code is A-12-323-13). The authors would like to thank Dr. Sina Andalib for his precious consults.
